# Future leaders in child and adolescent mental health research: addressing career challenges of young researchers across the WHO European Region

**DOI:** 10.1093/eurpub/ckag024

**Published:** 2026-02-02

**Authors:** Anastasia Giannaki, Syed Muhammad Aqeel Abidi, Anouk Boonstra, Hannah Brunskill, Francesco Andrea Causio, Theodoros Filippou, Anastasia Gatopoulou, Maria Gogou, Sophie Mae Harrington, Panagiota Kalpaxi, Jasmine Lee, Eleni Maousidi, Mohammad Shafi Mohammadi, Lotenna Olisaeloka, Christos Papaioannou, Raffaella Sibilio, Milica Sušić, Jennifer Hall, Joao Breda

**Affiliations:** WHO Quality of Care and Patient Safety Office, WHO Regional Office for Europe,Athens, Greece; PGME, The Aga Khan University Medical College Pakistan, Karachi, Pakistan; Department of Psychiatry and Neuropsychology, Maastricht University, Maastricht, the Netherlands; WHO Quality of Care and Patient Safety Office, WHO Regional Office for Europe,Athens, Greece; Department of Life Sciences and Public Health, Universitá Cattolica del Sacro Cuore, Rome, Italy; Medical School, University of CyprusNicosia, Cyprus; WHO Quality of Care and Patient Safety Office, WHO Regional Office for Europe,Athens, Greece; 2nd Pediatric Department, University General Hospital AHEPA, Thessaloniki, Greece; Psychology Department, University of Galway, Galway, Ireland; WHO Quality of Care and Patient Safety Office, WHO Regional Office for Europe,Athens, Greece; Centre for Longitudinal Studies, Social Research Institute, London, United Kingdom; WHO Quality of Care and Patient Safety Office, WHO Regional Office for Europe,Athens, Greece; Department of Psychology, Woman Online University (WOU), Afghanistan; Department of Psychiatry, School of Population and Public Health, Vancouver, Canada; Faculty of Medicine, University of Crete, Heraklion, Greece; WHO Quality of Care and Patient Safety Office, WHO Regional Office for Europe,Athens, Greece; Department of Law, CORE,Belgrade, Serbia; WHO Quality of Care and Patient Safety Office, WHO Regional Office for Europe,Athens, Greece; WHO Quality of Care and Patient Safety Office, WHO Regional Office for Europe,Athens, Greece

In the ongoing effort to strengthen leadership capacity in public health, a critical question emerges: Who gets to lead—and what stands in their way? While much attention is given to established leaders, less is said about the early-career researchers who will shape the future of the field. Their leadership potential is immense—but often stifled by systemic obstacles encountered early in their journey [1].

To delve deeper into these challenges, the WHO Young Researchers Forum launched a 2024 survey inviting early-career researchers to share their lived experiences. Respondents from diverse backgrounds revealed persistent barriers to entry, growth, and leadership. This commentary amplifies their collective voice and calls for action to ensure that future academic, research, and public health leaders are not lost to today’s systemic challenges.

## The WHO Young Researchers Forum: empowering the next generation

As part of its work to improve the quality of child and adolescent mental health care, the WHO Regional Office for Europe launched the WHO Young Researchers Forum (YRF) in 2024 to empower early-career professionals—aged 18–30—working in child and adolescent mental health research. A unique feature of the YRF is its wide reach: at the time of writing, there were 684 participants from 21 countries across the Region. The YRF aims to build a vibrant and resilient research ecosystem by fostering interdisciplinary and intra-country collaboration, enhancing data quality, and promoting innovative, real-world solutions.

By offering mentorship, peer support, and the opportunity to engage directly with WHO and policymakers across the Region, the YRF supports a new generation of researchers who are not only passionate about mental health but also equipped to inform real-life change [2]. In 2024, as part of the Forum’s ongoing determination to amplify young voices, a short survey was conducted to better understand the challenges faced by early-career researchers across the Region. The insights presented here reflect both their challenges and aspirations—and highlight the systemic changes needed to foster their full leadership potential.

## Career instability and leadership insecurity

A central theme across responses was a deep sense of career uncertainty. Many young researchers spoke of unstable positions, lack of career clarity, and difficulty transitioning into leadership roles. “Not knowing how to get my foot in the door into international public health is the most discouraging,” one respondent shared. Others noted that even high academic performance and motivation were not enough to secure sustainable career paths. Nearly all respondents described access to research funding as either “somewhat difficult” or “very difficult” to obtain. Without certain funding, emerging researchers struggle to focus on long-term goals or develop the soft skills needed for leadership—like policy engagement, team coordination, and communication skills. One participant put it simply: “I want to grow as a leader, but I’m too busy surviving.”

These concerns are echoed in the wider literature. Research shows that even highly capable early-career researchers often face unstable career prospects due to short-term contracts, unclear success metrics, and limited access to long-term funding or leadership pathways [3]. In the UK, for example, the success rate for early-career fellowship grants has declined from 19% to 12% in recent years [4]. These structural challenges feed into what has been called a “broken pipeline,” where early-career researchers struggle to transition into stable, long-term functions, including future leadership roles [5].

## Mentorship gaps and missed opportunities

Leadership is an essential professional quality, but it does not develop in isolation. Yet many respondents reported feeling unsupported and disconnected from mentorship networks. Some had informal advisors, but most lacked structured guidance or access to leaders who could model and support their development. When asked what would help, young researchers envisioned mentorship programs that were intentional, diverse, and cross-border. One suggestion: “A 1-year program where early-career researchers are matched with international mentors—with specific goals and feedback cycles”; another called for “a peer group to share experiences and learn from others—not just a top-down approach.”

Such findings are aligned with broader observations that poor mentorship remains a barrier for early-career researchers [4]. The absence of these systems deprives young researchers not just of knowledge but also of a sense of belonging and confidence, both of which are vital to stepping into leadership.

## Structural barriers to growth

Beyond mentorship and funding, respondents cited broader structural issues that hinder leadership development. These included lack of visibility in high-level discussions: many felt excluded from decision-making forums, limiting their exposure and influence. Others described limited opportunities for meaningful authorship or project leadership, sharing that they struggled to attain significant roles in publications and projects. Poor work–life balance was also a common theme, with long hours, short contracts, and pressure to “publish or perish” contributing to stress and burnout. Many respondents pointed to inequitable access to publication and networking opportunities, saying they had fewer chances to publish or present internationally. Even when researchers were optimistic about their career direction—many hoped to pursue PhDs or work in humanitarian leadership—the path to those goals felt disconnected from reality. Institutions, they noted, often celebrate leadership ideals but fail to create or fund environments where young professionals can safely build and practice those skills.

These concerns are echoed in the wider literature. Studies have shown that many PhD students and postdoctoral researchers experience high levels of anxiety and depression due to the structural pressures of academia [6]. Additionally, there are documented inequities in authorship representation in studies conducted in low- and middle-income countries, reflecting persistent barriers to participation and leadership in global research [7].

## A blueprint for action

Enhancing leadership skills in public health begins with addressing the barriers that prevent young professionals from developing them. Based on insights from the YRF survey, we propose four key actions:

1. Create structured, equitable leadership development programs for early-career researchers—including mentorship, project ownership, and real-time feedback.
2. Develop and expand opportunities for seed funding and fellowships that prioritize skill-building and allow space for long-term growth.
3. Integrate leadership and soft skills training into academic and research curricula from the postgraduate level onward.
4. Promote mental well-being and inclusive environments, where leadership is defined by support, vision, and care, and strong researcher–public health links turn evidence into meaningful policy and action.

These measures are not privileges; they are the foundations upon which future public health leadership is built.

## Conclusion

Despite coming from different countries, young researchers face similar challenges. They are not only future leaders but also current contributors whose voices, skills, and insights are essential to the present and future of public health. Yet without addressing the instability, invisibility, and inequity they face, we risk leaving behind a generation that has the passion to lead but lacks the opportunity.

If we are committed to enhancing leadership skills in public health, then we must first create systems where leadership can be learned, practiced, and sustained—beginning with those at the start of their journey. In the field of child and adolescent mental health, this is especially critical: persistent research gaps and limited leadership pathways risk stalling progress. Empowering young researchers equitably across countries to lead, shape, and translate evidence into practice is not only an investment in their potential; it is essential to improving the quality of care and mental health outcomes for children and adolescents.

## Data Availability

No new data were generated or analysed in support of this research.
